# Ultrasound and elastography role in pre- and post-operative evaluation of median neuropathy in patients with carpal tunnel syndrome

**DOI:** 10.3389/fneur.2022.1079737

**Published:** 2022-12-16

**Authors:** Han Wu, Hong-Juan Zhao, Wei-Li Xue, Yi-Chun Wang, Wu-Yue Zhang, Xiao-Lei Wang

**Affiliations:** In-Patient Ultrasound Department, Second Affiliated Hospital of Harbin Medical University, Harbin, China

**Keywords:** carpal tunnel syndrome, acoustic radiation force impulse, shear wave elastography, Boston Carpal Tunnel Questionnaire, cross-sectional area

## Abstract

**Introduction:**

Carpal tunnel syndrome (CTS) is a common compression neuropathy of the median nerve in the wrist. Early diagnosis of CTS is essential for selecting treatment options and assessing prognosis. The current diagnosis of CTS is based on the patient's clinical symptoms, signs, and an electromyography (EMG) test. However, they have some limitations. Recently, ultrasound has been adopted as an adjunct diagnostic tool for electromyography (EMG). Ultrasound is a non-invasive and cost-effective technique. It provides a dynamic display of morphological changes in the median nerve and an assessment of CTS etiology such as tenosynovitis, mass compression, and tendon disease. This study aimed to investigate the value of conventional ultrasound and real-time shear wave elastography (SWE) in evaluation of median neuropathy in patients with carpal tunnel syndrome (CTS) before and after surgery.

**Methods:**

First, the Boston Carpal Tunnel Questionnaire (BCTQ) was administered to patients with CTS. All subjects were measured at three levels: the distal 1/3 of the forearm, the carpal tunnel inlet, and the distal carpal tunnel using conventional ultrasound and SWE. Median nerve parameters were examined in patients with CTS 1 week after surgery.

**Results:**

The cross-sectional area (CSA) and stiffness of the median nerve at the carpal tunnel inlet and distal carpal tunnel were significantly higher in patients with CTS than in healthy controls (*p* < 0.001). The CSA and stiffness of the median nerve at the carpal tunnel inlet were statistically significantly significantly between pre- and postoperative patients with CTS (*p* < 0.001). The CSA and stiffness of the nerve in patients with CTS had a positive correlation with electrophysiology severity.

**Conclusions and discussion:**

Conventional ultrasound and elastography are valuable in the diagnosis of CTS and are useful in the clinical assessment of patient's nerve recovery after operation.

## 1. Introduction

Carpal tunnel syndrome (CTS) is a common compression neuropathy of the median nerve in the wrist. It accounts for 90% of all entrapment neuropathies, with a prevalence rate of approximately 3.8% in the population (1). Nerve damage can be reversed with early treatment and diagnosis to prevent permanent damage. Early diagnosis of CTS is essential for selecting treatment options and assessing prognosis (2, 3). The current diagnosis of CTS is based on the patient's clinical symptoms, signs, and an electromyography (EMG) test. However, symptoms and signs in patients with mild disease are unclear and lack objective judgment criteria (4). Electromyography is the gold standard for CTS diagnosis and yields 98% specificity and 75% sensitivity. This examination has some limitations, including being time-consuming, invasive, and has a 10–25% false-negative rate. In addition, it cannot directly assess the anatomical relationship between the median nerve and surrounding tissues (4–6). Certain imaging examinations are significant in CTS diagnosis. According to Park et al. (7), magnetic resonance imaging (MRI) can be a diagnostic tool for median neuropathy. This technology can measure the median nerve cross-sectional area (CSA), despite the procedure being expensive and time-consuming. Also, computed tomography (CT) can diagnose CTS by showing changes in the density of the compressed median nerve and occupying lesions (8, 9).

Recently, ultrasound has been adopted as an adjunct diagnostic tool for electromyography (EMG). Ultrasound is a non-invasive and cost-effective technique. It provides a dynamic display of morphological changes in the median nerve and an assessment of CTS etiology such as tenosynovitis, mass compression, and tendon disease (10–12). Previous studies have focused on the CSA of the median nerve at the carpal tunnel entrance in CTS patients with sensitivity and specificity comparable to electromyography examinations (13–16). On the contrary, shear-wave elastography (SWE) is a new technique for the quantitative measurement of tissue stiffness (17). It is a complement to conventional ultrasound in the assessment of neuropathy (18–21). Studies have reported the significance of elastography in the assessment of CTS (22–25). However, to the extent of our knowledge, there are few reports on the evaluation of pre- and post-operative changes in nerve stiffness in patients with CTS. Therefore, the purpose of this study was to investigate the value of conventional ultrasound and SWE on the median nerve in patients with CTS before and after surgery.

## 2. Materials and methods

The study was approved by the Ethics Committee of the Second Affiliated Hospital of Harbin Medical University. All examinations were conducted after written informed consent was obtained from all subjects.

### 2.1. Patients

In total, 50 wrists of 25 healthy volunteers and 54 wrists of 30 patients who underwent carpal tunnel release surgery were studied from October 2020 to June 2021. Inclusion criteria were as follows: patients who reported abnormal sensation or pain in their extremities and had a positive electromyography examination (26). Exclusion criteria were as follows: age < 18 years, history of previous hand surgery, and other conditions affecting the measurement of the median nerve. The scale used to measure the severity of CTS was as follows: negative (grade 0), mild (grade 1), moderate (grade 2), severe (grade 3), and extremely severe (grade 4) according to the results of electromyography. Questionnaires were administered to patients with CTS, and basic information was recorded for all participants.

### 2.2. Questionnaires

The Boston Carpal Tunnel Questionnaire (BCTQ) consists of two self-rating scales: the Symptom Severity Scale (SSS) and the Functional Status Scale (FSS) (27). High scores indicated more severe disease conditions. It was used to assess all patients with CTS.

### 2.3. Ultrasound examination

All examinations were performed using a 4C15 MHz linear array transducer (Aixplorer; Supersonic Imagine, Aix en Provence, France). All patients were placed in a seated position, facing the examiner, with the forearm fully exposed, the elbow bent at 90°, and the hand kept in a resting position. The median nerve CSA and nerve flattening rate (NFR) were measured at three sites: the distal 1/3 of the forearm, the carpal tunnel inlet (at the level of the doughy bone), and the distal carpal tunnel (at the level of the hook bone). NFR is the ratio of the transverse diameter of the nerve to the anterior-posterior diameter when the median nerve is compressed by entrapment. It is an ultrasound parameter to determine if the nerve is compressed. The mean value was calculated by taking the average of the replicate measurements. The transducer was rotated 90° to obtain a longitudinal image, and the image was frozen after obtaining the ideal image. SWE was activated at the same three loci as the 2D ultrasound. Three regions of interest (ROI) with a diameter of 1 mm were selected to calculate the average value. Each locus was measured in triplicate. During the measurement, a sufficient coupling agent was applied, and the probe was placed gently on the skin to avoid pressure. A week after the surgery, the conventional ultrasound and SWE were repeated on the same patients. The above operations and image analysis were performed by a qualified ultrasound physician with over 10 years of research in musculoskeletal aspects.

### 2.4. Statistical analyses

Data were analyzed using the SPSS version 23.0 (SPSS Inc., Chicago, USA) and MedCalc 20.0 software (Ostend, West-Vlaanderen, Belgium). Continuous variables were expressed as mean ± standard deviation (SD). The Kolmogorov-Smirnov test was used to analyze the normal distribution. Mann–Whitney U test was used to analyze the non-normally distributed measures. The correlation was expressed by Spearman's correlation coefficient. Receiver operating characteristic (ROC) curves for CSA and elasticity were obtained to calculate sensitivity, specificity, accuracy, the area under the curve (AUC), and optimal cutoff values. Z-test was used for AUC. Statistical significance was considered at *p* < 0.05.

## 3. Results

### 3.1. Study demographics

The demographic data for the patients are summarized in Table 1. There was no statistical difference in gender, age, and body mass index (BMI) between patients with CTS and healthy controls at *p* > 0.05. BCTQ scores (SSS and FSS) between pre- and postoperative groups with CTS did not show any significant statistical difference (*p* > 0.05). In total, six wrists (11.1%) reported negative electromyography results. Grading CTS by severity showed mild in 15 (27.8%), moderate in 18 (33.3%), severe in 13 (24.1%), and extremely severe in 2 (3.7%) cases with a positive neurophysiological result.

**Table 1:** Characteristics of patients.

	**Control group**	**CTS group**		***p-*value**
	**(*n* = 50)**	**(*****n*** = **54)**		
Age (y)^**#**^	55.84 ± 7.59	53.57 ± 8.72		0.400
Sex				0.299
Female	42 (84)	48 (89)		
Male	8 (16)	6 (11)		
BMI (kg/m^2^)^**#**^	22.22 ± 1.75	22.31 ± 1.98		0.670
BCTQ sore^**#**^		Preoperative group	Postoperative group	
SSS	/	4.87 ± 3.30	4.81 ± 3.22	0.257
FSS	/	2.20 ± 1.91	2.11 ± 1.75	0.132

CTS, carpal tunnel syndrome; BMI, body mass index; BCTQ, The Boston Carpal Tunnel Questionnaire; SSS, Symptom Severity Scale; FSS, Functional Status Scale. ^#^Data are means ± standard deviations.

### 3.2. Conventional ultrasound and SWE parameters in healthy controls and preoperative groups

The distal 1/3 of the forearm in the preoperative group was not statistically different at *p* > 0.05 in CSA, flattening rate, and E_mean_ of the median nerve compared to the healthy control group. However, the CSA, flattening rate, and E_mean_ of the median nerve at the carpal tunnel inlet and the distal carpal tunnel in the preoperative group were significantly higher than in healthy controls at *p* < 0.001 (Table 2).

**Table 2:** Conventional ultrasound and shear wave elastography parameters of the control group and the preoperative group.

	**Control group (*n* = 50)**	**Preoperative group (*n* = 54)**	***p-*value**
**Distal 1/3 of the forearm**			
CSA (mm^2^)^**#**^	5.95 ± 0.75	5.96 ± 0.83	0.661
NFR^#^	1.78 ± 0.34	1.73 ± 0.29	0.470
E _mean_ (kPa)^**#**^	25.38 ± 3.99	25.87 ± 4.81	0.271
**Carpal tunnel inlet**			
CSA (mm^2^)^**#**^	5.91 ± 0.90	16.00 ± 5.63	0.000*
NFR^#^	2.51 ± 0.48	3.11 ± 0.53	0.000*
E _mean_ (kPa)^**#**^	35.25 ± 23.38	188.21 ± 53.09	0.000*
**Distal carpal tunnel**			
CSA (mm^2^)^**#**^	4.86 ± 0.88	8.70 ± 2.48	0.000*
NFR^#^	2.05 ± 0.44	2.67 ± 0.78	0.000*
E _mean_ (kPa)^**#**^	27.83 ± 9.75	113.48 ± 51.74	0.000*

CSA, cross-sectional area; NFR, neural flattening rate; E_mean_, the mean shear wave velocity of the nerve. ^#^Data are means ± standard deviations. ^*^Indicates statistically significant difference.

### 3.3. Conventional ultrasound and SWE parameters between pre- and postoperative CTS groups

There was no significant difference in CSA, flattening rate, and E_mean_ of the median nerve at the distal 1/3 of the forearm (*p* > 0.05). The CSA, flattening rate, and E_mean_ of the median nerve at the carpal tunnel inlet and distal carpal tunnel were significantly lower in the postoperative group than in the preoperative group (*p* < 0.05) (Table 3).

**Table 3:** Conventional ultrasound and shear wave elastography parameters of the preoperative group and the postoperative group.

	**Preoperative group (*n* = 19)**	**Postoperative group (*n* = 19)**	***p-*value**
**Distal 1/3 of the forearm**			
CSA (mm^2^)^**#**^	6.51 ± 1.59	6.23 ± 0.84	0.954
NFR^#^	1.76 ± 0.25	1.71 ± 0.21	0.327
E _mean_ (kPa)^**#**^	25.45 ± 4.99	23.54 ± 3.67	0.133
**Carpal tunnel inlet**			
CSA (mm^2^)^**#**^	14.89 ± 4.25	11.63 ± 3.69	0.000*
NFR^#^	3.06 ± 0.51	2.44 ± 0.46	0.001*
E _mean_ (kPa)^**#**^	185.20 ± 34.97	67.46 ± 21.19	0.000*
**Distal carpal tunnel**			
CSA (mm^2^)^**#**^	8.44 ± 2.75	8.02 ± 2.90	0.389
NFR^#^	2.68 ± 0.56	2.34 ± 0.71	0.049*
E _mean_ (kPa)^**#**^	102.32 ± 37.61	34.88 ± 15.78	0.000*

CSA, cross-sectional area; NFR, neural flattening rate; E_mean_, the mean shear wave velocity of the nerve. ^#^Data are means ± standard deviations. ^*^Indicates statistically significant difference.

### 3.4. Conventional ultrasound and SWE parameters of healthy controls and postoperative CTS groups

The postoperative group had a significantly lower CSA and E_mean_ of the median nerve at the carpal tunnel inlet and the distal carpal tunnel than the healthy controls (*p* < 0.05) (Table 4).

**Table 4:** Conventional ultrasound and shear wave elastography parameters of the control group and the postoperative group.

	**Control group (*n* = 50)**	**Postoperative group (*n* = 19)**	***p*-value**
**Distal 1/3 of the forearm**			
CSA(mm^2^)^**#**^	5.95 ± 0.75	6.23 ± 0.84	0.597
NFR^**#**^	1.78 ± 0.34	1.71 ± 0.21	0.498
E _mean_ (kPa)^**#**^	25.38 ± 3.99	23.54 ± 3.67	0.712
**Carpal tunnel inlet**			
CSA(mm^2^)^**#**^	5.91 ± 0.90	11.63 ± 3.69	0.000*
NFR^**#**^	2.51 ± 0.48	2.44 ± 0.46	0.577
E_mean_ (kPa)^**#**^	35.25 ± 23.38	67.46 ± 21.19	0.000*
**Distal carpal tunnel**			
CSA(mm^2^)^**#**^	4.86 ± 0.88	8.02 ± 2.90	0.000*
NFR^**#**^	2.05 ± 0.44	2.34 ± 0.71	0.127
E _mean_ (kPa)^**#**^	27.83 ± 9.75	34.88 ± 15.78	0.041*

CSA, cross-sectional area; NFR, neural flattening rate; E_mean_, the mean shear wave velocity of the nerve. ^#^Data are means ± standard deviations. ^*^Indicates statistically significant difference.

### 3.5. Diagnostic performance of conventional ultrasound and SWE parameters

The CSA and E_mean_ at the carpal tunnel inlet and the distal 1/3 of the forearm showed good diagnostic performance (0.999, 0.993, 0.999, 0.992, *p* < 0.001). In this study, the corresponding cutoff value of CSA (8.2 mm^2^) was used to diagnose CTS, with a sensitivity of 96.2% and a specificity of 100% at the carpal tunnel inlet. We chose the optimal cutoff value of E_mean_ (102.9 Kpa) to diagnose CTS, with a sensitivity of 96.3% and a specificity of 98%. The difference in AUC between CSA and E_mean_ for CTS diagnosis was not statistically significant (Z = 1.15, *p* = 0.25). The CSA cutoff value (1.68 mm^2^) of the carpal tunnel inlet and the distal 1/3 of the forearm produced sensitivity and specificity of 86.6 and 100%, respectively. However, the cutoff value (3.72 mm^2^) of the E_mean_ ratio of the carpal tunnel inlet and the distal 1/3 of the forearm produced a sensitivity of 96% and a specificity of 96% (Table 5).

**Table 5:** AUC, sensitivity, specificity, and optimal cut-off values of nerve parameters for the diagnosis of CTS.

	**Sensitivity (%)**	**Sensitivity (%)**	**AUC**
Carpal tunnel inlet CSA≥8.2 mm^2^	96.2	100	0.999
Inlet/ distal 1/3 of the forearm CSA ratio≥1.68	86.8	100	0.999
Carpal tunnel inlet E _mean_≥102.9kPa	96.3	98	0.993
Inlet/ distal 1/3 of the Forearm E _mean_ ratio≥3.72	96	96	0.992

CSA, cross-sectional area; E_mean_, the mean shear wave velocity of the nerve. Data were tested with the area under the receiver operating characteristic (ROC) curve.

### 3.6. Correlation of electrophysiology severity with CSA and E_*mean*_

Electrophysiological severity had a positive correlation with CSA (*r*^2^ = 0.93, *p* < 0.001), whereas electrophysiological severity had a moderate correlation with E_mean_ (*r*^2^ = 0.60, *p* < 0.001).

## 4. Discussion

Studies suggest high pressure in the carpal tunnel to be the cause of CTS (28). The increased pressure affects the microcirculation in the median nerve, leading to edema and demyelinating lesions. Hence, there is a thickening of the nerve bundle and vessel walls (29, 30). Nerve entrapment injury causes intraneural ischemia, which leads to abnormal vascular endothelial permeability and manifests as intra-nerve edema and increased CSA measurement (29, 31). High-frequency ultrasound can detect changes in the median nerve, such as hypoechoic nerve, blurred nerve bundle, and increased cross-sectional area.

Byra et al. (32) reported variability in the echogenicity and shape of the median nerve in CTS and healthy patients. Also, the CSA of patients with CTS was significantly higher than healthy individuals (33). Notably, these findings were consistent with our study.

Furthermore, we found that the lack of differences between CSA at the distal carpal tunnel pre- and postoperatively could be attributed to delayed decompression.

We also found that the injury to the nerve did not involve the distal 1/3 of the forearm, and because of this, we could use the CSA at the distal 1/3 of the forearm as a reference value in this experiment to obtain a truncated value with less individual differentiation compared with the CSA at other locations.

Compression of microcirculation in the median nerve leads to focal demyelination and axonal degeneration with a fibrotic response (14, 30). Wang et al. (34) documented a relationship between carpal tunnel pressure and shear wave velocity. In our study, the cutoff values of the median nerve at the carpal tunnel inlet were higher than in other studies. We concede that participants had moderate and severe CTS with a higher degree of neuropathy, thus leading to a higher E_mean_.

**Figure 1:**
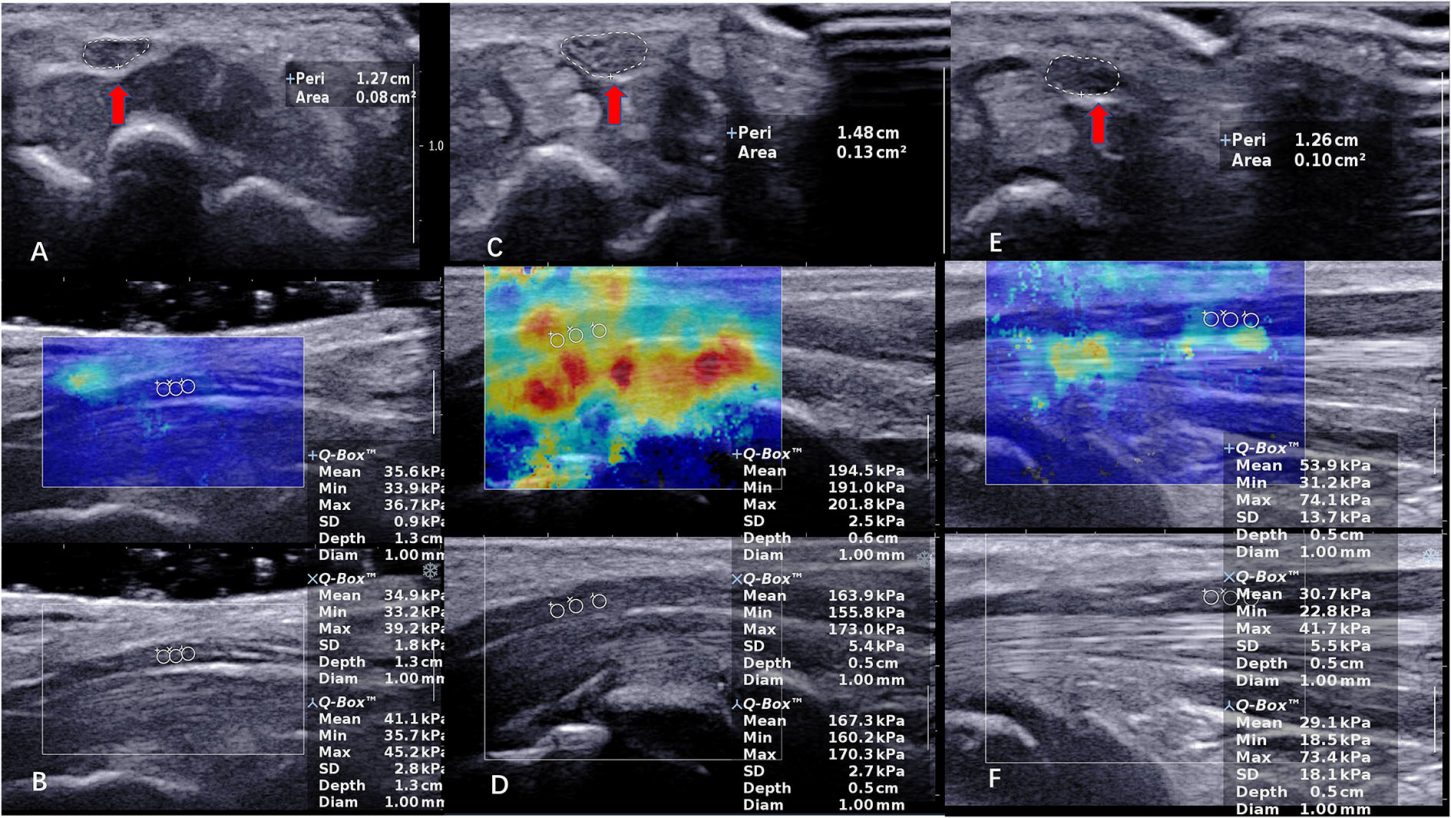
The CSA and SWE of the median nerve (MN) in a 40-year-old female control subject at carpal tunnel inlet. **(A,B)** (CSA = 0.08 cm^2^, E_mean_ = 37.2 kPa). The CSA and SWE of the median nerve (MN) in a 51-year-old female patient with severe CTS at carpal tunnel inlet in preoperative. **(C,D)** (CSA = 0.13 cm^2^, E_mean_ = 175.3 kPa). The CSA and SWE of the median nerve (MN) in a 51-year-old female patient with severe CTS at carpal tunnel inlet in postoperative. **(E,F)** (CSA = 0.10 cm^2^, E_mean_ = 37.9 kPa). The red arrow indicates the CSA of the nerve.

In our study, we found that the variance of E_mean_ at the carpal tunnel inlet and outlet in all populations is very high compared to 1/3 of the distal forearm (Figure 1). We hypothesized that there are more bones in the carpal tunnel inlet and outlet, which cause the “bone-proximity” hardening artifacts. These artifacts occur when the nerve travels near a bone plane that prevents the shear wave from homogeneously propagating at depth, thus causing local stress inhomogeneity. As Bortolotto reported, the stiffness of the median nerve progressively increases in its distal portions, where the nerve approaches the bone surface (35).

The correlation of electrophysiology severity with CSA and Emean is consistent with the results of Choi et al. (36) and Drakopoulos et al. (37). Therefore, we suggest that it is feasible to determine the severity of the disease in patients with CTS based on conventional ultrasound and SWE. Conversely, another study showed that nerve stiffness was a better predictor of disease severity than CSA, due to substantial axonal loss in extremely severe cases, which produces less swelling of the compressed nerve than in moderately severe cases (38).

There were two cases of extreme severity in our study that had no significant difference in assessing CTS severity. The quantitative evaluation of the median nerve injury in patients by conventional ultrasound and SWE had high diagnostic values (Table 5). Although there were no significant differences between the two techniques, the application of the SWE technique did not improve the diagnostic efficacy of CTS. Kantarci et al. (28) and Arslanet et al. (39) documented similar findings.

Currently, the best treatment for CTS is carpal tunnel release. Approximately, 70– 90% of patients undergo carpal tunnel dissection to release the median nerve and achieve satisfactory results (40, 41). The questionnaires can be used as a method to assess postoperative recovery (42). Compared to the BCTQ score, ultrasound better reflects the recovery of neuropathy before the symptoms improve.

The reduction in CSA and elasticity is due to the successful severance of the transverse carpal ligament, which releases nerve compression and reduces nerve edema. In our study, the differences in CSA and E_mean_ between postoperative patients with CTS and volunteers were statistically significant (Table 4). It can be attributed to the presence of severe permanent structural changes in the median nerve in some patients, as reported by Inui et al. (40). In addition, neurological symptoms were not completely relieved because of the short recovery time.

The differences in CSA of the median nerve measured at the carpal tunnel and proximal levels provided higher diagnostic efficacy than a single CSA measure (43). Scholars have suggested the use of different elasticity ratios to reduce individual variability and stiffness (13, 33, 44) due to the variability of CSA and elasticity in patients of different ages, weights, and genders.

In this experiment, we used CSA and E_mean_ of the nerve at the distal 1/3 of the forearm as internal control parameters. We analyzed the diagnostic performance of the CSA ratio and E_mean_ of the nerve at the carpal tunnel inlet and the distal 1/3 of the forearm. The ratio had a high diagnostic efficacy; this strategy increased the assessment efficiency and sensitivity of median nerve injury (45) but was not optimized by applying CSA or E_mean_ alone due to the low number of patients.

This study has some limitations: (a) A single-center study with a small sample size, (b) a preliminary study of postoperative nerve recovery in the short term; in future studies, we will refine the records of longer follow-ups. (c) We detected microvascular changes within the median nerve in some moderate to severe patients, which we believe is also a sign of pathological changes. However, we did not conduct further analysis. (d) The pressure applied to the surface with the probe cannot be controlled completely at the carpal tunnel inlet and outlet. Therefore, the recommendations for future research are to (a) increase the number of patients with mild and extremely severe CTS, (b) increase the patient follow-up information to assess long-term nerve recovery and explore the potential correlations between SWE parameters and clinical score in a later stage after surgery, (c) examine the microvascular anatomy of the median nerve in all patients in future studies, and (d) add an ultrasound gel pad to reduce excessive pressure when measuring the elasticity of the superficial nerve.

## 5. Conclusion

Conventional ultrasound and shear wave elastography have a strong correlation with clinical symptom scores and electromyography severity grading. This allowed the assessment of changes in postoperative nerve stiffness, which assisted in the clinical judgment of postoperative nerve recovery in patients with CTS.

## Data availability statement

The original contributions presented in the study are included in the article/supplementary material, further inquiries can be directed to the corresponding author.

## Ethics statement

The studies involving human participants were reviewed and approved by the Ethics Committee of the Second Affiliated Hospital of Harbin Medical University. The patients/participants provided their written informed consent to participate in this study.

## Author contributions

Conception and design of the study: X-LW. Ultrasound data acquisition: HW. Analysis of data: W-LX, Y-CW, and W-YZ. Drafting the manuscript: HW and H-JZ. Revising and final approval of the version to be published: X-LW. All authors contributed to the article and approved the submitted version.
